# 2-(5-Isopropyl-3-methyl­sulfanyl-1-benzofuran-2-yl)acetic acid

**DOI:** 10.1107/S1600536809021242

**Published:** 2009-06-10

**Authors:** Hong Dae Choi, Pil Ja Seo, Byeng Wha Son, Uk Lee

**Affiliations:** a Department of Chemistry, Dongeui University, San 24 Kaya-dong Busanjin-gu, Busan 614-714, Republic of Korea; b Department of Chemistry, Pukyong National University, 599-1 Daeyeon 3-dong, Nam-gu, Busan 608-737, Republic of Korea

## Abstract

There are two mol­ecules in the asymmetric unit of the title compound, C_14_H_16_O_3_S. In the crystal structure, the carboxyl groups are involved in inter­molecular O—H⋯O hydrogen bonds, which link the mol­ecules into centrosymmetric dimers. These dimers are further packed into stacks along the *a* axis by aromatic π–π inter­actions between the furan rings of adjacent mol­ecules [centroid–centroid distance = 3.430 (4) Å] and by additional C—H⋯π and C—H⋯O inter­actions.

## Related literature

For the crystal structures of similar 2-(3-methyl­sulfanyl-1-benzofuran-2-yl)acetic acid derivatives, see: Choi *et al.* (2009 ▶); Seo *et al.* (2007 ▶). For the biological and pharmacological activity of benzofuran compounds, see: Howlett *et al.* (1999 ▶); Ward (1997 ▶).
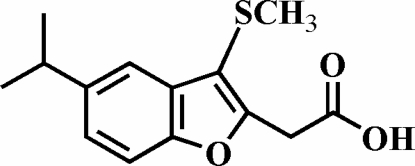


## Experimental

### 

#### Crystal data

C_14_H_16_O_3_S
*M* *_r_* = 264.33
Monoclinic, 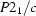

*a* = 17.160 (2) Å
*b* = 8.7773 (7) Å
*c* = 17.819 (2) Å
β = 93.905 (2)°
*V* = 2677.6 (5) Å^3^
*Z* = 8
Mo *K*α radiation
μ = 0.24 mm^−1^
*T* = 173 K
0.30 × 0.20 × 0.20 mm

#### Data collection

Bruker SMART CCD diffractometer
Absorption correction: none
19182 measured reflections
4727 independent reflections
3284 reflections with *I* > 2σ(*I*)
*R*_int_ = 0.066

#### Refinement

*R*[*F* ^2^ > 2σ(*F* ^2^)] = 0.052
*wR*(*F* ^2^) = 0.139
*S* = 1.09
4727 reflections
336 parameters
H atoms treated by a mixture of independent and constrained refinement
Δρ_max_ = 0.35 e Å^−3^
Δρ_min_ = −0.24 e Å^−3^

### 

Data collection: *SMART* (Bruker, 2001 ▶); cell refinement: *SAINT* (Bruker, 2001 ▶); data reduction: *SAINT*; program(s) used to solve structure: *SHELXS97* (Sheldrick, 2008 ▶); program(s) used to refine structure: *SHELXL97* (Sheldrick, 2008 ▶); molecular graphics: *ORTEP-3* (Farrugia, 1997 ▶) and *DIAMOND* (Brandenburg, 1998 ▶); software used to prepare material for publication: *SHELXL97*.

## Supplementary Material

Crystal structure: contains datablocks global, I. DOI: 10.1107/S1600536809021242/wm2239sup1.cif

Structure factors: contains datablocks I. DOI: 10.1107/S1600536809021242/wm2239Isup2.hkl

Additional supplementary materials:  crystallographic information; 3D view; checkCIF report

## Figures and Tables

**Table 1:** Hydrogen-bond geometry (Å, °)

*D*—H⋯*A*	*D*—H	H⋯*A*	*D*⋯*A*	*D*—H⋯*A*
O2—H2*O*⋯O3^i^	0.86 (4)	1.79 (4)	2.649 (3)	174 (4)
O5—H5*O*⋯O6^ii^	0.75 (4)	1.88 (4)	2.621 (3)	170 (5)
C19—H19⋯O5^iii^	0.95	2.70	3.567 (4)	151
C9—H9*B*⋯*Cg*4	0.99	2.57	3.350 (4)	136
C23—H23*A*⋯*Cg*2	0.99	2.58	3.299 (4)	129

Symmetry codes: (i) 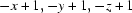; (ii) 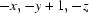; (iii) 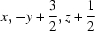. *Cg*2 and *Cg*4 are the centroids of the C2–C7 and C16-C21 benzene rings, respectively.

**Fig. 1.**
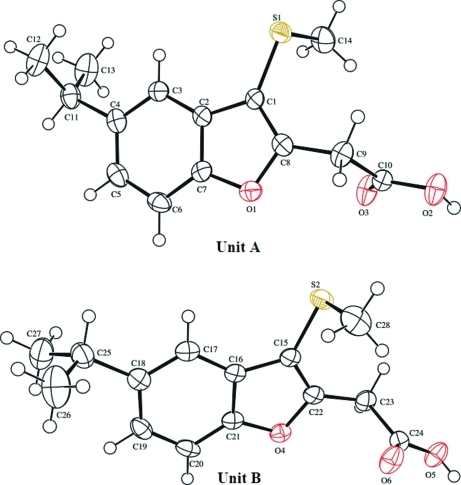
The molecular structure of the title compound with the atom numbering scheme. Displacement ellipsoids are drawn at the 50% probability level. H atoms are presented as a small cycles of arbitrary radius.

**Fig. 2.**
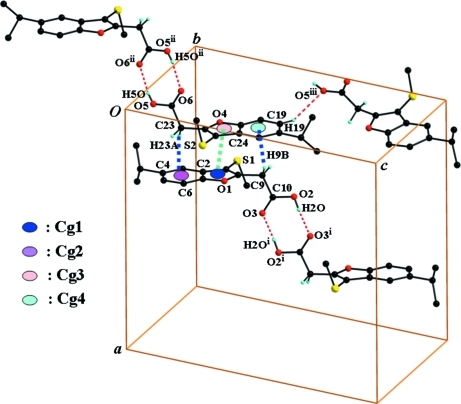
The O–H···O hydrogen bonds, and π–π, C–H···π, C–H···O interactions (dotted lines) in the title compound. [Symmetry code: (i) -x + 1, -y + 1, -z + 1; (ii) -x, -y + 1, -z; (iii) x, -y + 3/2, z + 1/2.]

## supplementary crystallographic information

### Comment

The benzofuran compounds have attracted considerable interest in view of their biological and pharmacological properties (Howlett *et al.*, 1999; Ward, 1997). As a part of our ongoing studies on the synthesis and structures of 2-(3-methylsulfanyl-1-benzofuran-2-yl)acetic acid analogues, the crystal structure of 2-(5-ethyl-3-methylsulfanyl-1-benzofuran-2-yl)acetic acid (Seo *et al.*, 2007) and 2-(3-methylsulfanyl-5-propyl-1-benzofuran-2-yl)acetic acid (Choi *et al.*, 2009) have been described in the literature. Here we report the crystal structure of the title compound, 2-(5-isopropyl-3-methylsulfanyl-1-benzofuran-2-yl)acetic acid which crystallizes with two unique molecules, denoted as A & B, in the asymmetric unit (Fig. 1).

The benzofuran unit is essentially planar, with a mean deviation of 0.003 (2) Å for A and 0.011 (2) Å for B, respectively, from the least-squares plane defined by the nine constituent atoms. In the crystal structure, the carboxyl groups are involved in intermolecular O–H···O hydrogen bonds (Fig. 2 and Table 1), which link the molecules into centrosymmetric dimers. These dimers are further packed into stacks along the *a*-axis by aromatic π–π interactions between the furan rings from the adjacent molecules. The *Cg*1···*Cg*3 distance is 3.430 (4) Å (*Cg*1 and *Cg*3 are the centroids of C1/C2/C7/O1/C8 furan ring and C15/C16/C21/O4/C22 furan ring, respectively). In addition, the crystal packing exhibits two different C–H···π interactions between a methylene H atom and the benzene ring from adjacent molecules (Table 1 and Fig. 2); *Cg*2 and *Cg*4 are the centroids of the C2–C7 benzene ring and the C16-C21 benzene ring, respectively, and a non-classical C–H···O hydrogen bond between a benzene H atom and the O atom of the hydroxy group (Table 1 and Fig. 2).

### Experimental

Ethyl 2-(5-isopropyl-3-methylsulfanyl-1-benzofuran-2-yl)acetate (292 mg, 1.0 mmol) was added to a solution of potassium hydroxide (337 mg, 6.0 mmol) in water (25 ml) and methanol (25 ml), and the mixture was refluxed for 5h, then cooled. Water was added, and the solution was extracted with dichloromethane. The aqueous layer was acidified to pH 1 with concentrated hydrochloric acid and then extracted with chloroform, dried over magnesium sulfate, filtered and concentrated under vacuum. The residue was purified by column chromatography (hexane-ethyl acetate,1 : 2 v/v) to afford the title compound as a colorless solid [yield 85%, m.p. 412-413 K; R_f_ = 0.69 (hexane-ethyl acetate, 1 : 2 v/v )]. Single crystals suitable for X-ray diffraction were prepared by evaporation of a solution of the title compound in benzene at room temperature. Spectroscopic analysis: ^1^H NMR (CDCl_3_, 400 MHz) δ 1.30 (d, J = 6.96 Hz, 6H), 2.33 (s, 3H), 3.01-3.07 (m, 1H), 4.03 (s, 2H), 7.19 (dd, J = 8.44 Hz and J = 1.84 Hz, 1H), 7.38 (d, J = 8.84 Hz, 1H), 7.47 (s, 1H), 10.08 (s, 1H); EI-MS 264 [M^+^].

### Refinement

Atoms H2O and H5O of the hydroxy groups were found in a difference Fourier map and refined freely. The other H atoms were positioned geometrically and refined using a riding model, with C–H = 0.95 Å for the aryl, 0.99 Å for the methylene, 1.00 Å for the methine, and 0.98 Å for the methyl H atoms.*U*iso(H) = 1.2*U*eq(C) for the aryl, methine and methylene H atoms, and 1.5*U*eq(C) for methyl H atoms.

### Crystal data

**Table:**

C_14_H_16_O_3_S	*F*(000) = 1120
*M**_r_* = 264.33	*D*_x_ = 1.311 Mg m^−^^3^
Monoclinic, *P*2_1_/*c*	Mo *K*α radiation, λ = 0.71073 Å
Hall symbol: -P 2ybc	Cell parameters from 5245 reflections
*a* = 17.160 (2) Å	θ = 2.4–28.0°
*b* = 8.7773 (7) Å	µ = 0.24 mm^−^^1^
*c* = 17.819 (2) Å	*T* = 173 K
β = 93.905 (2)°	Block, colorless
*V* = 2677.6 (5) Å^3^	0.30 × 0.20 × 0.20 mm
*Z* = 8	

### Data collection

**Table:**

Bruker SMART CCD diffractometer	3284 reflections with *I* > 2σ(*I*)
Radiation source: fine-focus sealed tube	*R*_int_ = 0.066
graphite	θ_max_ = 25.0°, θ_min_ = 1.2°
Detector resolution: 10.0 pixels mm^-1^	*h* = −20→20
φ and ω scans	*k* = −10→10
19182 measured reflections	*l* = −21→20
4727 independent reflections	

### Refinement

**Table:**

Refinement on *F*^2^	Primary atom site location: structure-invariant direct methods
Least-squares matrix: full	Secondary atom site location: difference Fourier map
*R*[*F*^2^ > 2σ(*F*^2^)] = 0.052	Hydrogen site location: difference Fourier map
*wR*(*F*^2^) = 0.139	H atoms treated by a mixture of independent and constrained refinement
*S* = 1.09	*w* = 1/[σ^2^(*F*_o_^2^) + (0.0613*P*)^2^ + 1.3164*P*] where *P* = (*F*_o_^2^ + 2*F*_c_^2^)/3
4727 reflections	(Δ/σ)_max_ < 0.001
336 parameters	Δρ_max_ = 0.35 e Å^−^^3^
0 restraints	Δρ_min_ = −0.24 e Å^−^^3^

### Special details

**Table:**

Geometry. All esds (except the esd in the dihedral angle between two l.s. planes) are estimated using the full covariance matrix. The cell esds are taken into account individually in the estimation of esds in distances, angles and torsion angles; correlations between esds in cell parameters are only used when they are defined by crystal symmetry. An approximate (isotropic) treatment of cell esds is used for estimating esds involving l.s. planes.
Refinement. Refinement of F^2^ against ALL reflections. The weighted R-factor wR and goodness of fit S are based on F^2^, conventional R-factors R are based on F, with F set to zero for negative F^2^. The threshold expression of F^2^ > 2sigma(F^2^) is used only for calculating R-factors(gt) etc. and is not relevant to the choice of reflections for refinement. R-factors based on F^2^ are statistically about twice as large as those based on F, and R- factors based on ALL data will be even larger.

### Fractional atomic coordinates and isotropic or equivalent isotropic displacement parameters (Å^2^)

**Table:**

	*x*	*y*	*z*	*U*_iso_*/*U*_eq_	
S1	0.37862 (4)	0.80938 (8)	0.21890 (4)	0.0331 (2)	
S2	0.12907 (4)	0.14800 (8)	0.26676 (5)	0.0351 (2)	
O1	0.33687 (10)	0.3877 (2)	0.28508 (10)	0.0279 (5)	
O3	0.46953 (11)	0.4873 (3)	0.40822 (11)	0.0375 (5)	
O2	0.41120 (12)	0.6069 (3)	0.50141 (12)	0.0371 (6)	
H2O	0.449 (2)	0.570 (5)	0.530 (2)	0.076 (14)*	
O4	0.15963 (10)	0.5807 (2)	0.21373 (11)	0.0284 (5)	
O6	0.02448 (11)	0.4616 (3)	0.09349 (11)	0.0416 (6)	
O5	0.09385 (13)	0.4311 (3)	−0.00875 (13)	0.0385 (6)	
H5O	0.063 (3)	0.459 (5)	−0.037 (2)	0.090 (18)*	
C1	0.36056 (14)	0.6137 (3)	0.22892 (15)	0.0225 (6)	
C2	0.35460 (14)	0.4999 (3)	0.16827 (15)	0.0230 (6)	
C3	0.36002 (15)	0.4995 (3)	0.08765 (16)	0.0261 (6)	
H3	0.3698	0.5919	0.0622	0.031*	
C4	0.35082 (15)	0.3625 (3)	0.04656 (16)	0.0289 (7)	
C5	0.33688 (16)	0.2281 (3)	0.08780 (18)	0.0332 (7)	
H5	0.3310	0.1346	0.0611	0.040*	
C6	0.33120 (16)	0.2260 (3)	0.16803 (17)	0.0333 (7)	
H6	0.3217	0.1342	0.1941	0.040*	
C7	0.34024 (14)	0.3644 (3)	0.20579 (15)	0.0250 (6)	
C8	0.34931 (14)	0.5411 (3)	0.29642 (16)	0.0248 (6)	
C9	0.34748 (15)	0.5971 (3)	0.37775 (15)	0.0287 (7)	
H9A	0.3422	0.7094	0.3768	0.034*	
H9B	0.3000	0.5555	0.3988	0.034*	
C10	0.41644 (15)	0.5572 (3)	0.43041 (15)	0.0244 (6)	
C11	0.35236 (17)	0.3567 (4)	−0.04190 (17)	0.0357 (7)	
H11	0.3469	0.2475	−0.0574	0.043*	
C12	0.28631 (18)	0.4423 (4)	−0.08121 (18)	0.0476 (9)	
H12A	0.2894	0.5498	−0.0665	0.057*	
H12B	0.2366	0.3994	−0.0672	0.057*	
H12C	0.2896	0.4338	−0.1357	0.057*	
C13	0.42626 (19)	0.4145 (5)	−0.07065 (19)	0.0541 (10)	
H13A	0.4703	0.3543	−0.0490	0.065*	
H13B	0.4335	0.5216	−0.0563	0.065*	
H13C	0.4236	0.4057	−0.1256	0.065*	
C15	0.14263 (14)	0.3457 (3)	0.26341 (15)	0.0248 (6)	
C16	0.14678 (14)	0.4533 (3)	0.32748 (15)	0.0227 (6)	
C17	0.14295 (15)	0.4436 (3)	0.40849 (16)	0.0288 (7)	
H17	0.1367	0.3472	0.4316	0.035*	
C18	0.14835 (16)	0.5757 (3)	0.45287 (17)	0.0303 (7)	
C19	0.15631 (15)	0.7170 (3)	0.41529 (17)	0.0322 (7)	
H19	0.1585	0.8077	0.4444	0.039*	
C20	0.16123 (16)	0.7293 (3)	0.33547 (18)	0.0323 (7)	
H20	0.1674	0.8253	0.3120	0.039*	
C21	0.15664 (14)	0.5951 (3)	0.29375 (16)	0.0250 (6)	
C22	0.15112 (14)	0.4274 (3)	0.19790 (16)	0.0263 (6)	
C23	0.15319 (15)	0.3839 (3)	0.11477 (16)	0.0312 (7)	
H23A	0.2005	0.4296	0.0952	0.037*	
H23B	0.1587	0.2718	0.1115	0.037*	
C24	0.08450 (15)	0.4305 (3)	0.06474 (16)	0.0262 (6)	
C25	0.14598 (19)	0.5682 (4)	0.54135 (18)	0.0416 (8)	
H25	0.1474	0.4582	0.5560	0.050*	
C26	0.0728 (2)	0.6345 (5)	0.5684 (2)	0.0606 (11)	
H26A	0.0275	0.5798	0.5453	0.073*	
H26B	0.0693	0.7424	0.5545	0.073*	
H26C	0.0736	0.6249	0.6233	0.073*	
C27	0.21352 (19)	0.6445 (5)	0.58342 (19)	0.0536 (10)	
H27A	0.2141	0.7528	0.5701	0.064*	
H27B	0.2623	0.5966	0.5702	0.064*	
H27C	0.2084	0.6340	0.6376	0.064*	
C28	0.02834 (17)	0.1368 (4)	0.2460 (2)	0.0464 (9)	
H28A	0.0014	0.1957	0.2832	0.070*	
H28B	0.0117	0.0301	0.2476	0.070*	
H28C	0.0153	0.1785	0.1957	0.070*	
C14	0.47646 (16)	0.8192 (4)	0.25281 (19)	0.0407 (8)	
H14A	0.4813	0.7860	0.3054	0.061*	
H14B	0.4950	0.9245	0.2494	0.061*	
H14C	0.5079	0.7528	0.2226	0.061*	

### Atomic displacement parameters (Å^2^)

**Table:**

	*U*^11^	*U*^22^	*U*^33^	*U*^12^	*U*^13^	*U*^23^
S1	0.0323 (4)	0.0251 (4)	0.0406 (5)	−0.0001 (3)	−0.0085 (3)	0.0003 (3)
S2	0.0312 (4)	0.0236 (4)	0.0487 (5)	0.0013 (3)	−0.0087 (3)	−0.0014 (3)
O1	0.0218 (10)	0.0294 (11)	0.0320 (11)	−0.0009 (8)	−0.0012 (8)	0.0058 (9)
O3	0.0219 (11)	0.0604 (15)	0.0296 (12)	0.0138 (10)	−0.0040 (9)	−0.0077 (10)
O2	0.0271 (11)	0.0563 (15)	0.0272 (12)	0.0128 (10)	−0.0046 (9)	−0.0069 (10)
O4	0.0240 (10)	0.0284 (11)	0.0325 (12)	−0.0012 (8)	−0.0002 (8)	0.0051 (9)
O6	0.0213 (11)	0.0693 (16)	0.0338 (12)	0.0094 (10)	−0.0006 (9)	−0.0044 (11)
O5	0.0249 (12)	0.0597 (16)	0.0307 (13)	0.0043 (10)	0.0001 (10)	0.0029 (11)
C1	0.0138 (12)	0.0261 (15)	0.0268 (16)	0.0015 (11)	−0.0037 (11)	−0.0003 (12)
C2	0.0116 (12)	0.0262 (15)	0.0307 (16)	0.0006 (11)	−0.0025 (11)	−0.0007 (12)
C3	0.0181 (13)	0.0282 (16)	0.0318 (17)	−0.0002 (11)	0.0002 (12)	0.0027 (13)
C4	0.0149 (13)	0.0359 (17)	0.0358 (17)	0.0032 (12)	0.0012 (12)	−0.0073 (14)
C5	0.0254 (15)	0.0276 (16)	0.046 (2)	0.0036 (12)	−0.0002 (14)	−0.0101 (14)
C6	0.0292 (16)	0.0230 (16)	0.047 (2)	0.0012 (12)	−0.0022 (14)	0.0032 (14)
C7	0.0143 (13)	0.0310 (16)	0.0292 (16)	0.0019 (11)	−0.0016 (11)	0.0034 (13)
C8	0.0139 (13)	0.0257 (15)	0.0343 (17)	0.0028 (11)	−0.0024 (11)	−0.0022 (12)
C9	0.0180 (14)	0.0401 (18)	0.0277 (16)	0.0056 (12)	−0.0008 (12)	0.0004 (13)
C10	0.0204 (14)	0.0287 (16)	0.0243 (16)	−0.0009 (12)	0.0027 (12)	0.0000 (12)
C11	0.0315 (16)	0.0401 (18)	0.0355 (18)	0.0025 (14)	0.0013 (13)	−0.0109 (15)
C12	0.0338 (18)	0.077 (3)	0.0315 (19)	0.0059 (17)	−0.0015 (14)	−0.0036 (17)
C13	0.0362 (19)	0.089 (3)	0.038 (2)	−0.0022 (19)	0.0099 (16)	−0.0099 (19)
C15	0.0141 (13)	0.0265 (15)	0.0331 (16)	0.0025 (11)	−0.0024 (11)	0.0008 (13)
C16	0.0091 (12)	0.0248 (15)	0.0339 (16)	0.0004 (10)	−0.0011 (11)	0.0015 (12)
C17	0.0212 (14)	0.0282 (16)	0.0371 (18)	−0.0008 (11)	0.0013 (12)	0.0053 (13)
C18	0.0217 (14)	0.0314 (17)	0.0377 (18)	0.0001 (12)	0.0020 (13)	−0.0014 (14)
C19	0.0241 (15)	0.0283 (16)	0.0438 (19)	0.0030 (12)	0.0005 (13)	−0.0100 (14)
C20	0.0277 (15)	0.0182 (15)	0.051 (2)	0.0005 (12)	−0.0010 (14)	0.0018 (13)
C21	0.0143 (13)	0.0262 (15)	0.0344 (17)	−0.0004 (11)	−0.0001 (11)	0.0042 (13)
C22	0.0130 (13)	0.0274 (15)	0.0379 (17)	0.0021 (11)	−0.0031 (12)	0.0002 (13)
C23	0.0212 (14)	0.0380 (18)	0.0341 (17)	0.0064 (12)	−0.0005 (12)	−0.0018 (14)
C24	0.0222 (15)	0.0285 (16)	0.0282 (17)	−0.0007 (12)	0.0038 (12)	−0.0030 (12)
C25	0.056 (2)	0.0343 (19)	0.0361 (19)	0.0008 (15)	0.0111 (16)	−0.0042 (15)
C26	0.044 (2)	0.088 (3)	0.052 (2)	−0.018 (2)	0.0202 (18)	−0.017 (2)
C27	0.043 (2)	0.078 (3)	0.039 (2)	0.0160 (19)	−0.0024 (16)	−0.0119 (19)
C28	0.0313 (17)	0.043 (2)	0.066 (2)	−0.0109 (15)	0.0088 (16)	−0.0017 (17)
C14	0.0276 (16)	0.047 (2)	0.048 (2)	−0.0114 (14)	0.0016 (14)	−0.0024 (16)

### Geometric parameters (Å, °)

**Table:**

S1—C14	1.747 (3)	C12—H12C	0.9800
S1—C1	1.757 (3)	C13—H13A	0.9800
S2—C28	1.745 (3)	C13—H13B	0.9800
S2—C15	1.752 (3)	C13—H13C	0.9800
O1—C8	1.377 (3)	C15—C22	1.387 (4)
O1—C7	1.432 (3)	C15—C16	1.480 (4)
O3—C10	1.188 (3)	C16—C21	1.398 (4)
O2—C10	1.347 (3)	C16—C17	1.452 (4)
O2—H2O	0.86 (4)	C17—C18	1.403 (4)
O4—C22	1.380 (3)	C17—H17	0.9500
O4—C21	1.436 (3)	C18—C19	1.421 (4)
O6—C24	1.212 (3)	C18—C25	1.581 (4)
O5—C24	1.330 (3)	C19—C20	1.435 (4)
O5—H5O	0.75 (4)	C19—H19	0.9500
C1—C8	1.386 (4)	C20—C21	1.392 (4)
C1—C2	1.470 (4)	C20—H20	0.9500
C2—C7	1.394 (4)	C22—C23	1.533 (4)
C2—C3	1.446 (4)	C23—C24	1.486 (4)
C3—C4	1.411 (4)	C23—H23A	0.9900
C3—H3	0.9500	C23—H23B	0.9900
C4—C5	1.419 (4)	C25—C26	1.493 (5)
C4—C11	1.579 (4)	C25—C27	1.495 (5)
C5—C6	1.440 (4)	C25—H25	1.0000
C5—H5	0.9500	C26—H26A	0.9800
C6—C7	1.392 (4)	C26—H26B	0.9800
C6—H6	0.9500	C26—H26C	0.9800
C8—C9	1.532 (4)	C27—H27A	0.9800
C9—C10	1.501 (4)	C27—H27B	0.9800
C9—H9A	0.9900	C27—H27C	0.9800
C9—H9B	0.9900	C28—H28A	0.9800
C11—C13	1.489 (4)	C28—H28B	0.9800
C11—C12	1.494 (4)	C28—H28C	0.9800
C11—H11	1.0000	C14—H14A	0.9800
C12—H12A	0.9800	C14—H14B	0.9800
C12—H12B	0.9800	C14—H14C	0.9800
			
C14—S1—C1	100.68 (14)	C21—C16—C17	119.6 (2)
C28—S2—C15	100.39 (14)	C21—C16—C15	103.7 (2)
C8—O1—C7	105.5 (2)	C17—C16—C15	136.6 (2)
C10—O2—H2O	110 (3)	C18—C17—C16	120.4 (3)
C22—O4—C21	106.2 (2)	C18—C17—H17	119.8
C24—O5—H5O	123 (4)	C16—C17—H17	119.8
C8—C1—C2	108.6 (2)	C17—C18—C19	117.4 (3)
C8—C1—S1	125.0 (2)	C17—C18—C25	121.5 (3)
C2—C1—S1	126.4 (2)	C19—C18—C25	121.1 (3)
C7—C2—C3	120.0 (2)	C18—C19—C20	123.2 (3)
C7—C2—C1	103.4 (2)	C18—C19—H19	118.4
C3—C2—C1	136.6 (2)	C20—C19—H19	118.4
C4—C3—C2	120.4 (3)	C21—C20—C19	117.4 (3)
C4—C3—H3	119.8	C21—C20—H20	121.3
C2—C3—H3	119.8	C19—C20—H20	121.3
C3—C4—C5	117.2 (3)	C20—C21—C16	121.9 (3)
C3—C4—C11	122.5 (3)	C20—C21—O4	126.9 (2)
C5—C4—C11	120.3 (3)	C16—C21—O4	111.2 (2)
C4—C5—C6	123.4 (3)	O4—C22—C15	110.4 (2)
C4—C5—H5	118.3	O4—C22—C23	115.6 (2)
C6—C5—H5	118.3	C15—C22—C23	134.0 (3)
C7—C6—C5	117.1 (3)	C24—C23—C22	116.2 (2)
C7—C6—H6	121.4	C24—C23—H23A	108.2
C5—C6—H6	121.4	C22—C23—H23A	108.2
C6—C7—C2	122.0 (3)	C24—C23—H23B	108.2
C6—C7—O1	126.2 (2)	C22—C23—H23B	108.2
C2—C7—O1	111.8 (2)	H23A—C23—H23B	107.4
O1—C8—C1	110.6 (2)	O6—C24—O5	125.3 (3)
O1—C8—C9	116.1 (2)	O6—C24—C23	118.1 (3)
C1—C8—C9	133.3 (3)	O5—C24—C23	116.6 (2)
C10—C9—C8	116.8 (2)	C26—C25—C27	107.7 (3)
C10—C9—H9A	108.1	C26—C25—C18	112.7 (3)
C8—C9—H9A	108.1	C27—C25—C18	114.2 (3)
C10—C9—H9B	108.1	C26—C25—H25	107.3
C8—C9—H9B	108.1	C27—C25—H25	107.3
H9A—C9—H9B	107.3	C18—C25—H25	107.3
O3—C10—O2	125.6 (3)	C25—C26—H26A	109.5
O3—C10—C9	120.2 (3)	C25—C26—H26B	109.5
O2—C10—C9	114.3 (2)	H26A—C26—H26B	109.5
C13—C11—C12	107.7 (3)	C25—C26—H26C	109.5
C13—C11—C4	113.9 (3)	H26A—C26—H26C	109.5
C12—C11—C4	112.8 (2)	H26B—C26—H26C	109.5
C13—C11—H11	107.4	C25—C27—H27A	109.5
C12—C11—H11	107.4	C25—C27—H27B	109.5
C4—C11—H11	107.4	H27A—C27—H27B	109.5
C11—C12—H12A	109.5	C25—C27—H27C	109.5
C11—C12—H12B	109.5	H27A—C27—H27C	109.5
H12A—C12—H12B	109.5	H27B—C27—H27C	109.5
C11—C12—H12C	109.5	S2—C28—H28A	109.5
H12A—C12—H12C	109.5	S2—C28—H28B	109.5
H12B—C12—H12C	109.5	H28A—C28—H28B	109.5
C11—C13—H13A	109.5	S2—C28—H28C	109.5
C11—C13—H13B	109.5	H28A—C28—H28C	109.5
H13A—C13—H13B	109.5	H28B—C28—H28C	109.5
C11—C13—H13C	109.5	S1—C14—H14A	109.5
H13A—C13—H13C	109.5	S1—C14—H14B	109.5
H13B—C13—H13C	109.5	H14A—C14—H14B	109.5
C22—C15—C16	108.5 (2)	S1—C14—H14C	109.5
C22—C15—S2	124.3 (2)	H14A—C14—H14C	109.5
C16—C15—S2	127.2 (2)	H14B—C14—H14C	109.5
			
C14—S1—C1—C8	75.9 (3)	C28—S2—C15—C22	−83.6 (3)
C14—S1—C1—C2	−104.3 (2)	C28—S2—C15—C16	96.2 (2)
C8—C1—C2—C7	−0.3 (3)	C22—C15—C16—C21	0.8 (3)
S1—C1—C2—C7	179.84 (19)	S2—C15—C16—C21	−178.99 (19)
C8—C1—C2—C3	179.8 (3)	C22—C15—C16—C17	−179.4 (3)
S1—C1—C2—C3	0.0 (4)	S2—C15—C16—C17	0.8 (4)
C7—C2—C3—C4	0.0 (4)	C21—C16—C17—C18	0.6 (4)
C1—C2—C3—C4	179.9 (3)	C15—C16—C17—C18	−179.1 (3)
C2—C3—C4—C5	−0.5 (4)	C16—C17—C18—C19	1.0 (4)
C2—C3—C4—C11	177.2 (2)	C16—C17—C18—C25	−178.8 (2)
C3—C4—C5—C6	0.6 (4)	C17—C18—C19—C20	−1.8 (4)
C11—C4—C5—C6	−177.1 (2)	C25—C18—C19—C20	178.0 (3)
C4—C5—C6—C7	−0.2 (4)	C18—C19—C20—C21	1.0 (4)
C5—C6—C7—C2	−0.3 (4)	C19—C20—C21—C16	0.7 (4)
C5—C6—C7—O1	−179.9 (2)	C19—C20—C21—O4	179.5 (2)
C3—C2—C7—C6	0.4 (4)	C17—C16—C21—C20	−1.5 (4)
C1—C2—C7—C6	−179.5 (2)	C15—C16—C21—C20	178.4 (2)
C3—C2—C7—O1	−180.0 (2)	C17—C16—C21—O4	179.5 (2)
C1—C2—C7—O1	0.1 (3)	C15—C16—C21—O4	−0.6 (3)
C8—O1—C7—C6	179.7 (3)	C22—O4—C21—C20	−178.7 (3)
C8—O1—C7—C2	0.1 (3)	C22—O4—C21—C16	0.3 (3)
C7—O1—C8—C1	−0.3 (3)	C21—O4—C22—C15	0.3 (3)
C7—O1—C8—C9	179.2 (2)	C21—O4—C22—C23	−179.5 (2)
C2—C1—C8—O1	0.4 (3)	C16—C15—C22—O4	−0.7 (3)
S1—C1—C8—O1	−179.74 (17)	S2—C15—C22—O4	179.11 (17)
C2—C1—C8—C9	−179.0 (3)	C16—C15—C22—C23	179.1 (3)
S1—C1—C8—C9	0.8 (4)	S2—C15—C22—C23	−1.1 (4)
O1—C8—C9—C10	73.6 (3)	O4—C22—C23—C24	−70.7 (3)
C1—C8—C9—C10	−107.0 (3)	C15—C22—C23—C24	109.6 (3)
C8—C9—C10—O3	1.0 (4)	C22—C23—C24—O6	−19.0 (4)
C8—C9—C10—O2	−178.9 (2)	C22—C23—C24—O5	162.5 (2)
C3—C4—C11—C13	59.0 (4)	C17—C18—C25—C26	−110.7 (3)
C5—C4—C11—C13	−123.4 (3)	C19—C18—C25—C26	69.5 (4)
C3—C4—C11—C12	−64.2 (4)	C17—C18—C25—C27	126.1 (3)
C5—C4—C11—C12	113.4 (3)	C19—C18—C25—C27	−53.7 (4)

### Hydrogen-bond geometry (Å, °)

**Table:**

*D*—H···*A*	*D*—H	H···*A*	*D*···*A*	*D*—H···*A*
O2—H2O···O3^i^	0.86 (4)	1.79 (4)	2.649 (3)	174 (4)
O5—H5O···O6^ii^	0.75 (4)	1.88 (4)	2.621 (3)	170 (5)
C19—H19···O5^iii^	0.95	2.70	3.567 (4)	151
C9—H9B···Cg4	0.99	2.57	3.350 (4)	136
C23—H23A···Cg2	0.99	2.58	3.299 (4)	129

Symmetry codes: (i) −*x*+1, −*y*+1, −*z*+1; (ii) −*x*, −*y*+1, −*z*; (iii) *x*, −*y*+3/2, *z*+1/2.
